# IgA Vasculitis: Etiology, Treatment, Biomarkers and Epigenetic Changes

**DOI:** 10.3390/ijms22147538

**Published:** 2021-07-14

**Authors:** Hitomi Sugino, Yu Sawada, Motonobu Nakamura

**Affiliations:** Department of Dermatology, University of Occupational and Environmental Health,1-1, Iseigaoka, Yahatanishi-Ku, Kitakyushu, Fukuoka 807-8555, Japan; hsugino@med.uoeh-u.ac.jp (H.S.); motonaka@med.uoeh-u.ac.jp (M.N.)

**Keywords:** IgA vasculitis, treatment, biomarker, epigenetic changes

## Abstract

IgA, previously called Henoch-Schönlein vasculitis, is an essential immune component that drives the host immune response to the external environment. As IgA has the unique characteristic of a flexible response to broad types of microorganisms, it sometimes causes an autoreactive response in the host human body. IgA vasculitis and related organ dysfunction are representative IgA-mediated autoimmune diseases; bacterial and viral infections often trigger IgA vasculitis. Recent drug developments and the presence of COVID-19 have revealed that these agents can also trigger IgA vasculitis. These findings provide a novel understanding of the pathogenesis of IgA vasculitis. In this review, we focus on the characteristics of IgA and symptoms of IgA vasculitis and other organ dysfunction. We also mention the therapeutic approach, biomarkers, novel triggers for IgA vasculitis, and epigenetic modifications in patients with IgA vasculitis.

## 1. Introduction

The human body is surrounded by the external environment. The skin is the outermost organ of the human body. It is exposed to various environmental factors, such as microorganisms, and drives the cutaneous immune response to protect the host human body. This self-defense action against the external environment sometimes triggers excessive inflammatory reactions, namely autoimmune reactions.

Immunoglobulin is a key driver of host defense immunity against microorganisms; there are several subtypes, including IgG, IgM, and IgA. Among other immunoglobulin subtypes, IgA has the unique characteristic of broad recognition of microorganisms. IgA sometimes causes autoimmune reactions, causing the immune complex to drive the inflammatory response in the host. IgA vasculitis, previously called Henoch–Schönlein vasculitis, is a representative autoimmune disease mediated by IgA deposition on the small blood vessels and causes inflammatory reactions in various organs. In this review, we focus on the basic characteristics of IgA, IgA vasculitis symptoms, therapeutic options, biomarkers, and epigenetic modifications.

## 2. Genetics

Advances in genetic analysis techniques have also been applied to elucidate the pathomechanism of IgA vasculitis [1,2].

López-Mejías et al. confirmed that IgA vasculitis is associated with human leukocyte antigen (HLA) class II, HLA-DRB1*01 allele in 342 Spanish patients with IgA vasculitis and 303 sex and ethnically matched controls. The HLA-DRB1*01 allele was found in 43% of patients with IgA vasculitis and in 7% of controls [3]. This was due to the increased frequency of the HLA-DRB1*0103 phenotype.

The same group also examined the samples of 349 Spanish patients with IgA vasculitis and 335 sex- and ethnically matched controls. They found a statistically significant increase in the HLA-B*41:02 allele in patients with IgA vasculitis when compared with controls, which was independent of the previously reported association with the HLA-DRB1*01 allele [4].

López-Mejías et al. performed the first genomewide association study (GWAS) using samples from 308 IgA vasculitis patients and 1018 healthy controls [5]. A linkage disequilibrium block of polymorphisms that maps to an intergenic region in human leukocyte antigen (HLA) class II, between HLA-DQA1 and HLA-DQB1, was strongly associated with susceptibility to IgA vasculitis.

Since 2002, there have been several reports on the association between IgA vasculitis and the polymorphisms of several genes other than HLA.

Amoli et al. examined 96 patients and 109 controls [6]. They found a significant association between carriage of IL-1 receptor antagonist allele 2 (ILRN*2) and severe renal involvement, manifested as nephrotic syndrome and/or renal insufficiency, and permanent renal involvement (renal sequelae).

Amoli et al. also found a significant association between IL-8 gene polymorphisms and renal involvement [7]. They found a significantly increased frequency of allele A of the IL-8 gene polymorphism in patients with IgA vasculitis who developed renal manifestations compared with patients without renal involvement.

The combination of genomic data of different pathologies as a single phenotype has emerged as a useful strategy to identify genetic risk loci shared among immune-mediated diseases. Carmona’s group have identified a new risk of allele A of IL-8 gene polymorphism on IgA vasculitis with renal involvement [8].

Taken together, although there have been many reports on the association of non-HLA genes with the risk or severity of IgA vasculitis, HLA genes are most associated with the risk of IgA vasculitis.

## 3. IgA Structure and Flexibility

### 3.1. The Structure of IgA

IgA acts in various mammals to protect against external microorganisms. IgA has two subtypes: IgA1 and IgA2. As a structural difference, IgA1 has an O-linked glycosylation rich structure in the hinge region. This unique structure of 13 amino acids extends in the hinge region longer than IgA2, suggesting that IgA1 broadly acts to recognize various antigens [9]; however, this site becomes a weak point of IgA1 because it is often the target of bacterial proteases [10]. IgA consists of two heavy chains and light chains that organize Fab regions with the domains of Cα1, VH1, VL, and CL, and are responsible for antigen recognition. Among these domains, Cα1 is a unique component of IgA [9]. The Fc region consists of two Cα2 and Cα3 domains. As one of the unique characteristics of IgA, this Fc region, namely the J chain, can bind to another IgA of the J chain to form a dimer immune complex [9]. 

Immunoglobulin is generally produced by B cells; however, IgA is produced by mucosal membranes or glands, such as salivary glands, sweat glands, and the gut, and its amount is greater than that of other types of immunoglobulin [9]. IgA binds to pathogens and viruses on the surface of the mucous membrane to prevent bacterial and viral infections. Furthermore, general immunoglobulins respond to specific bacteria and viruses [11]; however, IgA has the capacity to respond to specific bacteria and viruses in addition to various non-specific types of pathogens [12,13,14]. 

### 3.2. The Flexibility of IgA

**Figure 1:**
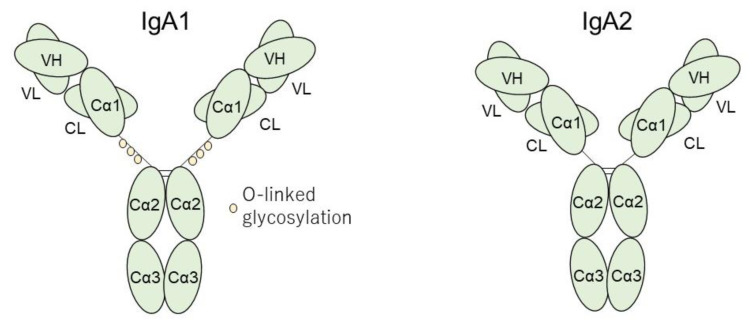
The structure of IgA1 and IgA2. IgA1 has an O-linked glycosylation rich structure in the hinge with 13 amino acids extension in the hinge region longer than IgA2. IgA consists of two heavy chains and light chains that organize Fab regions with the domains of Cα1, VH1, VL, and CL, and are responsible for antigen recognition. Cα1 is a unique component in IgA. The Fc region consists of two Cα2 and Cα3 domains, namely J chain, which bind to another IgA of the J chain to make a dimer immune complex.

Regarding structural aspects, Cα1 is a specific Fab domain in IgA, and other immunoglobulins do not exhibit this domain [9] (Figure 1). This specific domain enables IgA to have greater variation in its response to pathogens. IgA also exhibits a hydrophobic site between the VH and Cα1 domains [15], and a hydrophobic structure is expected to contribute to protein–protein interactions, including antigen–antibody reactions [16,17]. IgA1 has an O-linked glycosylation rich structure in the hinge region and the modification of this site contributes to an increased affinity to antigen [18], suggesting that this variable function in the hinge region of IgA1 contributes to the antigen reactive variation in IgA.

IgA is divided into classical and natural IgA [13]. Classical IgA is a T cell-dependent immunoglobulin that responds to specific pathogens with high affinity and antigen specificity [13]. During the maturation of germinal centers in the lymph nodes, IgA-producing B cells are selected based only on high affinity for pathogens and not specificity [19]. In contrast, natural IgA is reactive to commensal bacteria in a T cell-independent manner with low affinity but broader recognition of commensal bacteria [20]. These two different actions of IgA against pathogens are important for host defense against pathogens. Indeed, IgA deficiency is known to result in insufficient host defense action against pathogens, leading to the frequent onset of infectious diseases, suggesting that the characteristics of high affinity but no high specific reaction of IgA play a crucial role in the host defense [21].

## 4. Triggers and Pathogenesis of IgA Vasculitis

### 4.1. Triggers of IgA Vasculitis 

As IgA is driven in response to external stimuli, especially microorganisms, it is assumed that various microorganisms have the capacity to induce abnormal IgA autoimmune reactions in the host. Consistently, many types of bacteria and viruses are thought to be associated with the pathogenesis of IgA vasculitis. The representative causative pathogens are *Streptococcus* [22,23,24], *S. aureus* [25,26,27,28], *Helicobacter pylori* [29,30], varicella-zoster virus [31,32], hepatitis virus [33], Parvovirus [34,35], human immunodeficiency virus [36], cytomegalovirus [37], and *Clostridium difficile* [38]. Therefore, these microorganisms are triggers for the development of IgA vasculitis.

### 4.2. The Pathogenesis of IgA Vasculitis

After creating IgA immunocomplexes and deposition on small vessels, IgA activates mannan-binding lectin and alternative complement pathways [39,40]. Aberrant IgA responses underlie the pathogenesis of IgA vasculitis. Especially, in IgA nephritis patients, IgA1 is aberrantly glycosylated, and the hinge-region O-linked glycans are galactose-deficient [41]. Galactose-deficient IgA1 shows a better binding efficacy to mesangial cells compared with normal glycosylated IgA1 [18], suggesting that the modification of the hinge region in IgA1 is key to inducing IgA-mediated vascular damage. In addition, the immune complex with IgA1 obtained from patients with IgA nephritis shows high affinity to mesangial cells [18], indicating that IgA has a broad affinity, possibly leading to an autoimmune reaction [42,43].

FcαRI is a receptor for IgA and this signal has a bifunctional action in both proinflammatory and anti-inflammatory effects [44,45,46]. FcαRI is expressed on monocytes, macrophages, intestinal dendritic cells, and Kupffer cells [46]. The induction of FcαRI signaling is associated with the immunoreceptor tyrosine-based activation motif (ITAM). The binding of complexed monomeric serum IgA Fc domains to the membrane distal domain of FcαRI initiates signal cascades to enhance the inflammatory response, while uncomplexed monomeric IgA associates with FcαRI, the ITAM inhibitory signal cascade is initiated to promote anti-inflammatory action. Consistently, excessive IgA immune complexes or IgA-opsonized bacteria drive FcαRI-mediated immune cell activation, resulting in severe tissue damage, as observed during chronic inflammation and autoimmunity [47]. In addition, FcαRI on Kupffer cells enhances the efficient phagocytosis of bacteria coated with serum IgA [48]. Interestingly, FcαRI expression is strictly regulated by cytokines, such as G-CSF [48]. IgA also enhances FcαRI-mediated LTB4 production, resulting in the enhancement of neutrophil migration in IgA-triggered immune complex deposition sites [49]. 

After inflammatory damage in blood vessels, transendothelial migration of neutrophils occurs. VEGF promotes vascular permeability and promotes inflammatory cell migration from the vessels [50] and is associated with the development of IgA vasculitis [51]. The immune complex also enhances C3 and C5 production in endothelial cells, inducing IL-8, E-selectin, and ICAM 1 production [52]. Infiltration of inflammatory cells also increased the expression of ICAM 1 [53].

The cytokine profiles in the sera are shown in Table 1. General proinflammatory and inflammatory cytokines are increased in IgA vasculitis and nephritis, such as IL-1β, IL-4, IL-6, IL-8, IL-12p70, IL-17A, TNF-α, and IFN-γ. IL-6 levels are also increased in patients with IgA colitis. These inflammatory cytokines are orchestrated during the development of IgA-mediated autoimmune reactions. However, there are some controversial results, especially for TNF-α. As shown in TNF inhibitor-mediated IgA vasculitis, it seems that the involvement of TNF is not simple in the pathogenesis of IgA vasculitis. In addition, IL-17 is upregulated in IgA vasculitis and nephritis; however, IL-17 inhibitors are also related to the occurrence of IgA vasculitis, suggesting that TNF and IL-17 are involved in the cytokine production mediated by IgA vasculitis.

In the current study, IgA1 was identified as an autoantibody in host immunity [61], especially in IgA nephritis. IgA1 triggers immune complex deposition in the blood vessels, leading to the activation of complement factors and the exacerbation of vascular inflammation and damage [9].

In IgA1, this unique O-linked carbohydrate site seems to be associated with the pathogenesis of IgA nephritis from the dominant deposition of galactose-deficient IgA compared with IgA vasculitis without nephritis and healthy subjects. Interestingly, there was no significant difference in galactose-deficient IgA1 deposition between healthy subjects and IgA vasculitis without nephritis [62], suggesting that there is a different IgA1 dominant deposition pattern in the skin and kidneys in patients with IgA vasculitis.

Fatty acids are involved in various inflammatory reactions in the human body. LTB4 is derived from omega-6 fatty acids and exerts inflammatory responses and neutrophil migration and activation. LTB4 is increased in patients with IgA vasculitis [63] and decreased after treatment, suggesting that LTB4 might play a role in the acceleration of IgA vasculitis in some parts. 

Platelet-activating factor (PAF) is a lipid mediator involved in several allergic diseases and is released from eosinophils, neutrophils, and mast cells. PAF is involved in IgA vasculitis [64] and promotes IgA production [65]. 

### 4.3. The Difference of IgA between Adults and Pediatrics

It remains unclear whether there is a clear difference in IgA levels between pediatric and adult patients. As IgA vasculitis and nephritis show different clinical courses between adult and pediatric patients, it is assumed that there are some differences in IgA between adult and pediatric patients. The first difference is the amount of IgA, which contributes to the development of IgA [47]. Consistently, IgA production in adults is much higher than in children [66]. For instance, 2–3-year-old and 5–6-year-old children showed IgA levels of 40.8% and 69.5%, respectively, compared with adults [66]. IgA levels reach adult proportions after the age of 11 years. These findings suggest that IgA levels in children might not be at a sufficient volume to respond to vessels and the kidneys. 

Another possibility is that the frequency of memory B cells might be a clue to answering this question. The frequency of memory B cells in the peripheral blood is higher in adults than in children [67]. Schoolchildren showed a decreased frequency of memory B cells compared to adults; however, adolescents showed almost the same level of the frequency of adult memory B cells, without significant differences. These findings suggest that pediatric memory B cells might show less activity in memorized IgA production, leading to continuous pathogenic IgA production.

The difference might also depend on other immune profile differences. In the comparison of IgA nephritis between pediatric and adult patients, CD68+ macrophages were increased in adult glomerular and interstitial sites in the kidney. Macrophage function is decreased in pediatric patients compared with adults, such as cytotoxicity [68] and anti-tumor immunity [69].

In contrast, galactose-deficient IgA in patients with pediatric IgA vasculitis and nephritis is similar to that in adults [70]. This finding suggests that pathogenic IgA1 might not be different between adult and pediatric IgA vasculitis and nephritis. In addition, there is no evidence of the difference in the survival duration of memory B cells between pediatric and adult patients, and the actual impact of the frequency of memory B cells remains unclear. Therefore, further investigation is necessary to clarify this basic question. 

### 4.4. The Relationship with Coronavirus Disease 2019 (COVID-19)

Interestingly, several studies have reported COVID-19 associated IgA vasculitis [71,72,73,74,75,76,77]. COVID-19 caused by the severe acute respiratory syndrome coronavirus 2 (SARS-CoV-2) and the COVID-19 pandemic is currently an ongoing global problem and is known to cause vasculitislike syndromes [78]. The characteristics of patients with IgA vasculitis following COVID-19 are summarized in Table 2. There were seven cases of IgA vasculitis. The median age was 23.3 years and the ratio of adults to children was 4:3. Interestingly, all cases were male. Other symptoms excluding cutaneous purpura were observed in three of seven cases with abdominal pain, and three of seven cases with nephritis. Furthermore, all nephritis cases were adults, and 75% of adult cases showed IgA vasculitis following COVID-19 infection, whereas IgA nephritis was not identified in the children. 

COVID-19 has the potential to induce a cytokine storm, which might also be involved in the pathogenesis of IgA vasculitis. In addition, as another possibility, the medications for COVID-19 are not completely excluded as a trigger, similar to drug-induced IgA vasculitis.

### 4.5. The Relationship with TNF or IL-17 Inhibitors

In the skin, the TNF and IL-17 pathways are involved in various inflammatory skin diseases. Psoriasis is a representative inflammatory skin disease mediated by inflammatory pathways; it causes scaly erythematous plaques and influences inflammation in various internal organs. Consistently, these cytokine-targeted treatments showed strong efficacy in psoriatic skin inflammation. A neutrophil-mediated inflammatory reaction is downstream of the TNF- and IL-17 mediated inflammatory pathways. Therefore, these cytokines are also thought to be associated with the pathogenesis of IgA vasculitis. On the contrary, several recent reports suggest a possible pathogenetic role of TNF-mediated immune reactions in IgA vasculitis. One study reported that two cases showed the deposition of IgA in the renal biopsy among 39 cases of vasculitis during treatment with TNF inhibitors [79]. Another study showed that six out of nine patients experienced IgA vasculitis onset during TNF inhibitors; however, three of five patients continued to use TNF inhibitors and fully recovered while maintaining TNF inhibitor treatment [80]. IL-17A inhibitors have also been reported as triggers of IgA vasculitis [81]. The detailed molecular mechanisms remain unclear and may be related to some paradoxical side effects of these cytokine-targeted inhibitor treatments, as seen in psoriasis dermatitis following these inhibitor treatments [82,83]. Table 1 shows that the fluctuation of TNF in patients with IgA vasculitis is controversial, suggesting that the involvement of TNF is not simple. Some complex cytokine pathways, such as IFN, might also be involved in TNF inhibitor-related IgA vasculitis. Further investigation will be desired to clarify the actual therapeutic efficacy for IgA vasculitis.

## 5. Symptoms

IgA vasculitis involves various small vessels as well as the skin, joints, gastrointestinal tract, and kidneys. The skin manifestation of IgA vasculitis is palpable purpura commonly distributed in the lower extremities and sometimes the upper limbs and trunk. Among the small vessels, the blood vessels in the skin are mainly affected, predominantly capillaries, venules, or arterioles [84]. The histology of this purpura exhibits nuclear dust, extravasation of erythrocytes, and inflammatory cell infiltration surrounding the affected vessels with IgA deposition. 

Approximately 65% of patients with IgA vasculitis experience abdominal pain [85]. In addition, 30% of patients experience gastrointestinal bleeding [85]. Gastrointestinal symptoms generally appear within 1 week after the onset of purpura in the skin [86].

Kidney involvement is the most important organ dysfunction in determining the therapeutic options for IgA vasculitis and influencing its prognosis. IgA vasculitis in adult patients showed a high prevalence of IgA nephritis, with a tendency to be severe compared with that in children [87].

## 6. Treatment

### 6.1. Corticosteroids

IgA vasculitis patients show significant improvement of cutaneous purpura symptoms with the treatment of prednisolone 1 mg/kg daily [88]; however, there was no significant difference in the recurrence rate even after the administration of corticosteroids [88]. Although prolonged cutaneous purpura cases tend to become a risk factor for renal dysfunction, there is no preventive efficacy for IgA nephritis in the early administration of corticosteroids [89]. In contrast, IgA nephritis is a therapeutic candidate for corticosteroid administration. The early administration of prednisolone contributes to improvement in the renal function [90].

### 6.2. Colchicine

Colchicine is an alkaloid extracted from the corm of the meadow saffron or autumn crocus [91] and has various anti-inflammatory actions. Colchicine suppresses superoxide production from neutrophils [92], neutrophil chemotaxis [93], and lysozyme production [94]. While no clinical trials have evaluated the therapeutic efficacy of colchicine for IgA vasculitis, there were two case studies reporting the efficacy of colchicine for IgA vasculitis. 

### 6.3. Dapsone

Dapsone is an antibiotic that inhibits bacterial synthesis of dihydrofolic acid by competing with para-aminobenzoate for the active site of dihydropteroate synthase, resulting in the inhibition of nucleic acid synthesis [95,96]. In addition, dapsone has anti-inflammatory effects, such as the suppression of IL-8, TNF, toxic-free radical production, and inflammatory cell migration. Therefore, these actions are thought to be a promising therapeutic option for refractory IgA vasculitis [97]. Dapsone also showed therapeutic action against cutaneous purpura in IgA vasculitis [98,99,100].

Previous case reports suggest a possible therapeutic potential of dapsone for IgA vasculitis [98,101]. A recent systematic review analysis identified that therapeutic response was obtained in 23.1% of patients during the initial 1–2 days and 65.4% of patients within 1 week [95].

### 6.4. Intravenous Immunoglobulin

Intravenous immunoglobulin is currently used for the treatment of Guillain–Barré syndrome, myasthenia gravis, and bullous diseases [102,103]. As the mechanism of therapeutic action of intravenous immunoglobulin, it can impair autoreactive T cell responses by blocking their interaction with antigen presentation cells [104], downregulating antibody production by B cells [105], and blocking Fc-receptor-mediated immune response [106]. Recent studies have identified the therapeutic potential of intravenous immunoglobulin for IgA vasculitis. 

One study showed that 11 patients with IgA nephropathy received high-dose immunoglobulins (2 g/kg body weight), and histological changes and renal function were evaluated [107]. Proteinuria and reduced glomerular filtration rate were impaired by intravenous immunoglobulin treatment. The staining intensities of IgA and C3 in glomerular deposition were also decreased by the treatment. In another study, 14 patients with IgA nephritis received intravenous immunoglobulin treatment, and 1 patient withdrew from the study [108]. This study showed significantly decreased proteinuria, serum IgA, and β2 microglobulin levels.

### 6.5. Rituximab

Rituximab is a monoclonal antibody against CD20, which is a representative surface cell marker on B cells and is expected to suppress functional pathogenic IgA-producing B cells in patients with IgA vasculitis. 

There are several clinical reports of rituximab-treated refractory IgA vasculitis [109,110]. A recent systematic review of 20 studies including 35 well-characterized IgA vasculitis treated with rituximab showed that 94.3% of patients exhibited clinical improvement and 74.3% of patients achieved remission [111].

### 6.6. Angiotensin-Converting-Enzyme Inhibitor

ACE inhibitors are used for the treatment of hypertension [112,113] and inhibit the activity of ACE, which is an important component of the renin–angiotensin system converting angiotensin I to angiotensin II [114], and hydrolyzes bradykinin [115], leading to lower arteriolar resistance and increased venous capacity. 

A randomized controlled trial of IgA nephritis patients followed up for 5 years investigated the long-term renal outcome of ACE inhibitor/angiotensin receptor antagonist therapy [116]. The treatment showed lower serum creatinine, lower proteinuria, and fewer numbers progressing to end-stage renal failure. Another randomized trial in IgA nephritis showed that a high-dose regimen of ACE inhibitor (losartan 200 mg/day) showed significantly higher eGFR, lower proteinuria, and impaired eGFR reduction.

However, there is no evidence of the anti-inflammatory action of ACE inhibitors. This treatment is a part of supportive therapy to reduce IgA nephritis progression, similar to many renal diseases including SLE and scleroderma-related renal disease. 

### 6.7. Omega-3 Fatty Acids

Omega-3 fatty acids are present in fish oils and nuts [117]. Omega-3 fatty acids and their metabolites have potential anti-inflammatory action in inflammatory skin diseases [117,118,119,120]. Purpura is not a typical form of inflammatory skin disease, but causes inflammation in the blood vessels. Several studies have evaluated the anti-inflammatory action and therapeutic potential of omega-3 fatty acids in IgA vasculitis and nephritis.

In one study conducted by Hamazaki et al., 20 patients were divided into 2 groups with or without fish oil treatment (1.6 g EPA and 1.0 g DHA per day) for 1 year and were evaluated for renal function in IgA nephritis. The fish oil-treated group maintained their renal function, whereas the control group showed worsening of renal function [121]. 

In another study, 14 patients receiving omega-3 fatty acids (0.85 g EPA and 0.57 g DHA) were compared with 14 patients in the control group. The omega-3 fatty acids treated group showed a mean annual change in serum creatinine levels and GFR, indicating a favorable response [122].

A multicenter, placebo-controlled, randomized trial was conducted to evaluate the efficacy of fish oil in patients with IgA nephropathy [123]. Fifty-five patients received 12 g of daily fish oil, and 51 patients received olive oil as a placebo. The annual median changes in serum creatinine concentrations in the fish oil group were lower than those in the placebo group. The cumulative percentage of patients who died or had end-stage renal disease was 40 percent in the placebo group and 10 percent in the fish oil group.

### 6.8. Immunosuppressive Agents

Immunosuppressive agents show multiple steps of immunosuppressive effects against inflammatory diseases and exhibit beneficial effects on inflammatory diseases. In IgA nephritis, cyclophosphamide treatment alone has no beneficial impact [124]; however, corticosteroid combination therapy is effective for IgA nephritis [125]. Azathioprine therapy also showed a therapeutic effect on IgA nephritis with a combination of corticosteroids [126].

### 6.9. Lectin Pathway Treatment

The lectin pathways mediate glomerular injury and contribute to the development of IgA nephritis [127]. Narsoplimab is a monoclonal antibody against mannan-binding lectin serine peptidase 2 (MASP-2), a key component of the lectin pathway. Only one case report showed that narsoplimab has therapeutic efficacy in refractory IgA nephritis [128].

## 7. Biomarker

Since the degree of vasculitis is sometimes difficult to evaluate on superficial physical examination of the skin, biomarkers are required to predict the progression of IgA vasculitis and the presence of nephritis and colitis. Several studies have investigated biomarkers to predict the severity of IgA vasculitis and other organ involvement.

As biomarkers for IgA vasculitis, the severity of IgA vasculitis is associated with neutrophil/lymphocyte rate [129], serum neopterin and ischemia-modified albumin levels [130], skin miRNA-223-3p expression [131], and serum and urine levels of NGAL, KIM-1, and L-FABP [132]. IgA vasculitis patients sometimes exhibit ANCA positivity; however, there were no significant differences in the disease-free survival rates of chronic kidney disease and end-stage renal disease between IgA vasculitis patients with and without ANCA [133].

As biomarkers for IgA nephritis, a systematic literature review was performed to predict the presence of IgA nephritis or determine its severity [134]. In urine samples, kidney injury molecule-1 (KIM-1), monocyte chemotactic protein-1 (MCP-1), and N-acetyl-β-glucosaminidase (NAG) correlate with the disease severity of nephritis. In addition, the presence of IgA nephritis is associated with serum Gd-IgA1 and urinary IgA, IgG, IgM, IL-6, IL-8, IL-10, and IgA-IgG and IgA-sCD89 complexes [54], neutrophil count and neutrophil/lymphocyte rate [135], and serum angiotensinogen concentration [136]. The acute phase of IgA vasculitis is related to the expression levels of miRNA-33 and miRNA-34 [137] and aPS/PT-positivity [138]. 

As biomarkers for colitis, neutrophil count and neutrophil/lymphocyte ratio are higher in IgA colitis [129,135]. IgA colitis is inversely correlated with the expression of miRNA-155-5p and miRNA-146a-5p in the skin [131]. 

## 8. Epigenetic Modification

Although a minority of DNA sequence alterations are not infrequent in humans, as exemplified by DNA insertions in HPV-and EBV-related cancers, the majority of DNA sequence information is not generally changed during one’s lifetime; however, the gene information is regulated by acquired chemical modifications of DNA and DNA-binding proteins, especially histones [139,140]. In general, DNA and DNA-binding proteins are in contact with each other to avoid excessive transcriptional activation. However, once DNA is released from histones, open chromatin is formed, followed by the activation of the transcription activation response to the appropriate external stimulation [139]. Recent studies have also identified that epigenetic modifications are also involved in the pathogenesis of IgA vasculitis.

IgA vasculitis patients exhibit impaired ERK signaling, which is related to the downregulation of the DNA methyltransferase DNMT1 [141]. The histone demethylase KDM4C was identified as a risk factor for IgA vasculitis [142]. The enhancement of histone modification of H3 acetylation in peripheral blood mononuclear cells was significantly increased in patients with IgA vasculitis and nephritis, and H3K4 methylation was also increased in patients with IgA nephritis. In addition, other epigenetic modification enzymes, HDAC1, 2, and 4 SIRT1, LSD1, and KDM3A are decreased, whereas CREBBP, PCAF, SETD1A, MLL, STEDB1, and SUV39H2 are increased in IgA vasculitis, suggesting that these epigenetic modification enzymes might organize DNA and histone modifications in patients with IgA vasculitis and exacerbate inflammatory responses [143].

## 9. Conclusions

This review updates the recent knowledge and current problems in IgA vasculitis. Although the actual etiology and triggers of IgA vasculitis remain unclear, the presence of IgA vasculitis following novel drug administration and COVID-19 infection might be helpful to gain a deeper understanding. As the deposition pattern of IgA subtypes seems to be different between IgA vasculitis and nephritis, there are some different molecular mechanisms that cause IgA deposition in the small vessels in different organs. Currently, there is no specific treatment for skin purpura. Therefore, it is desirable to identify a novel therapeutic approach for skin eruptions because this is often a refractory and long-lasting form of skin inflammation. In addition, the knowledge of epigenetic modifications in IgA vasculitis is currently being developed. This information will be helpful in gaining a better understanding of the pathogenesis of IgA vasculitis. 

## Figures and Tables

**Table 1:** Cytokine profiles in IgA vasculitis, nephritis, and colitis.

	IgA Vasculitis	IgA Vasculitis Nephritis	IgA Colitis
IL-1β	↑ [54] → [55,56]	↑ [55] → [54,57]	No report
IL-2	→ [58]	→ [58]	No report
IL-4	↑ [58]	↑ [58]	No report
IL-6	↑ [54,56,58,59] → [55]	↑ [54,55,57,58] → [60]	↑ [60]
IL-8	↑ [55,56]	↑ [55,60]	→ [60]
IL-9	→ [56]	No report	No report
IL-10	→ [55,56] ↓ [58]	→ [55,56] ↓ [58]	No report
IL-12p70	→ [54,55]	→ [54,55]	No report
IL-17A	↑ [58]	↑ [58]	No report
IL-23	→ [56]	No report	No report
TNF-α	↑ [56] → [54,55] ↓ [58]	→ [54,55,60] ↓ [58]	→ [60]
IFN-γ	↑ [58]	↑ [58] → [57]	No report

**Table 2:** The summary of cases of IgA vasculitis following COVID-19.

Author	Age	Sex	Days after COVID-19 Test Positive	Involvement	Treatment
Suso, et al. [71]	78	Male	5 weeks later	Skin Nephritis	Steroid pulse plus Rituximab
Hoskins, et al. [72]	2	Male	Same time	Skin Abdominal pain	Intravenous steroid
Allez et al. [73]	24	Male	Unknown	Skin Abdominal pain	Methylprednisolone 0.8 mg/day
Sandhu et al. [74]	22	Male	Same time	Skin Nephritis	Prednisolone 1 mg/kg
AlGhoozi et al. [75]	4	Male	37 days later	Skin	Not described
Jacobi, et al. [76]	3	Male	Same time	Skin Abdominal pain	Antibiotic
Li et al. [77]	30	Male	Same time	Skin Nephritis	Losartan 25 mg following prednisolone 40 mg for 7 days

## Data Availability

Not applicable.
